# Tailoring of a Smartphone Smoking Cessation App (Kick.it) for Serious Mental Illness Populations: Qualitative Study

**DOI:** 10.2196/14023

**Published:** 2019-09-03

**Authors:** Pauline Klein, Sharon Lawn, George Tsourtos, Joep van Agteren

**Affiliations:** 1 Flinders Human Behaviour and Health Research Unit, Department of Psychiatry Flinders University Adelaide Australia; 2 College of Medicine and Public Health Flinders University Adelaide Australia; 3 Wellbeing and Resilience Centre South Australian Health and Medical Research Institute Adelaide Australia

**Keywords:** mental health, mHealth, tobacco, smoking cessation, public health, technology

## Abstract

**Background:**

Smoking rates of Australians with severe mental illness (SMI) are disproportionately higher than the general population. Despite the rapid growth in mobile health (mHealth) apps, limited evidence exists to inform their design for SMI populations.

**Objective:**

This study aimed to explore the feasibility, acceptability, and utility of adapting a novel smoking cessation app (Kick.it) to assist smokers with SMI to prevent smoking relapse and quit.

**Methods:**

Using co-design, two in-depth interviews with 12 adult smokers and ex-smokers with SMI were conducted in this qualitative study. Stage 1 interviews explored participants’ smoking-related experiences and perceptions of social support for smoking cessation, informed the development of the stage 2 interview schedule, and provided context for participants’ responses to the second interview. Stage 2 interviews explored participants’ perceptions of the feasibility, utility, and acceptability of the app features for SMI populations.

**Results:**

People with SMI perceived mHealth interventions to support their quit smoking attempts as feasible, acceptable, and useful. Key emerging themes included personalization of the app to users’ psychosocial needs, a caring app to mediate self-esteem and self-efficacy, an app that normalizes smoking relapse and multiple quit attempts, a strong focus on user experience to improve usability, and a social network to enhance social support for smoking cessation.

**Conclusions:**

This study gained an in-depth understanding of the lived experiences of smoking and quitting among people with SMI and their perception of the Kick.it app features to help inform the tailoring of the app. Specific program tailoring is required to assist them in navigating the complex interactions between mental illness and smoking in relation to their psychosocial well-being and capacity to quit. This study describes the adaptations required for the Kick.it app to meet the specific needs and preferences of people with SMI. Results of this study will guide the tailoring of the Kick.it app for SMI populations. The study findings can also inform a co-design process for the future development and design of smoking cessation apps for SMI populations.

## Introduction

### Background

Tobacco smoking is a major cause of preventable mortality and morbidity [1] and health inequalities for people with severe mental illness (SMI), including extreme social, economic, and physical health disadvantages [2]. SMI diagnoses include, for example, schizophrenia and bipolar disorder. In Australia, the smoking rates for SMI populations have remained inequitably high [3] for more than a decade [4]. Smoking rates for SMI populations in South Australia are alarmingly high, at almost triple (43.6%), that of the general population (15%) [5]. The high prevalence of smoking in this population is indicative of mental illness and smoking being intrinsically linked [4,6]. This is evident as people with mental illness often identify as smokers [6] and use smoking as a form of self-medication to help them cope with and relieve their symptoms of mental illness [4,6,7]. Owing to the inseparable nature of mental illness and smoking, nicotine addiction in this population has been difficult to treat [4].

Smoking-related studies have revealed that most people with SMI want to quit [8] and often attempt to quit but can find it challenging to quit without support [4,9]. A review of smoking cessation interventions, such as motivational interviewing, found limited evidence to support the interventions’ effectiveness in assisting people with schizophrenia to quit [10]. Cutting-edge digital health technology, such as mobile health (mHealth) smoking cessation apps [11-13], may contribute to the solutions needed to address this significant public health problem [11,14].

There are currently hundreds of smoking cessation apps available for download; however, limited studies have been conducted to assess the quality of app design. Research assessing the quality of generic smoking cessation apps revealed that most do not adhere to best-practice guidelines for smoking cessation, such as recommending pharmacotherapy [15,16]. Many smoking cessation apps also rated low on technical quality [17]. A review of 112 smoking cessation apps found that only 6 of these apps rated high on technical quality, such as having aesthetic appeal [16]. Despite the lack of quality for the vast majority of apps, scientific studies on smartphone interventions are promising in increasing cessation. Smokers who received the smoking cessation interventions demonstrated as much as a 1.7 times higher quit rate than smokers who did not receive the interventions [18].

In contrast to the vast availability of generic smoking cessation apps, there are only 3 that have been tailored for SMI populations [19-21]. This highlights a substantial gap in the availability of smoking cessation apps for SMI populations. This is particularly important when considering the cognitive impairments many people with SMI have to endure [22], which limits their ability to use apps [15,23,24]. A study on the QuitPal app developed by the National Cancer Institute, found that people with SMI experienced problems navigating the app, such as entering data, which was particularly relevant among participants with cognitive impairments and tremors [25]. Vilardaga et al used co-design, a person-centered approach to technology design, to involve people with SMI in the tailoring of the Learn to Quit app, developed by the University of Washington. Findings indicated that adapting a user experience (UX) approach with simple functionality, including large buttons and simple screens, improved the utility, usability, and acceptability of the app among people with SMI [21]. UX is a human-centered approach to improving end users’ performance and their psychological experience of technology systems [26]. Furthermore, exploration of participants’ preferences for app features found that people with SMI were interested in gamification (application of game design components and game principles in nongame systems) [27], interactive strategies to develop quit skills, and tracking devices for monetary incentives [21,25].

Building on this existing knowledge, there is a need to gain a deeper understanding of the relationship between mental illness, smoking, and smoking cessation to inform the design of effective smoking cessation app approaches for this population. This can help to guide how an app can be tailored to meet the specific requirements of people with SMI to reduce their smoking and quit. Currently, there are no smoking cessation apps for SMI populations that have been investigated within an Australian context, regarding their acceptability, feasibility, and usefulness. This study aims to address these important issues in relation to tailoring the Kick.it app for SMI populations [14].

### The Kick.it App and Its Theoretical Frameworks

Kick.it is a generic Australian-based prototype app that was originally co-designed for use by the general population of smokers [28], using intervention mapping (IM), which is a rigorous multitheoretical intervention development framework [29]. This consisted of a comprehensive needs analysis of the literature and stakeholder input from health professionals and smokers to identify the problem behaviors and determinants for smoking cessation. A co-design principle has also been used in this study to tailor the app for SMI populations before releasing the app on the marketplace. The design of the app for SMI populations enables app users to create a profile (ie, input information about their psychiatric diagnoses and smoking) and receive a personalized quit program that offers smoking cessation approaches tailored to meet their unique needs. These smoking cessation approaches are based on multitheoretical perspectives [28], as follows.

The Theoretical Domains Framework, a valid multitheoretical approach [30], underpinned the determinants for smoking cessation (eg, knowledge and skills) and the change objectives required to assist app users to quit (eg, increased knowledge of and ability to implement quit strategies) [28]. The Behavior Change Technique Taxonomy (v1) was used to identify behavior change approaches [31] and behavior change outcomes. These were then translated into app features and practical applications for smoking cessation [28]. The Persuasive System Design, a framework for technology development that targets attitude and behavior change, was also applied to inform the choice of app features [32].

**Figure 1:**
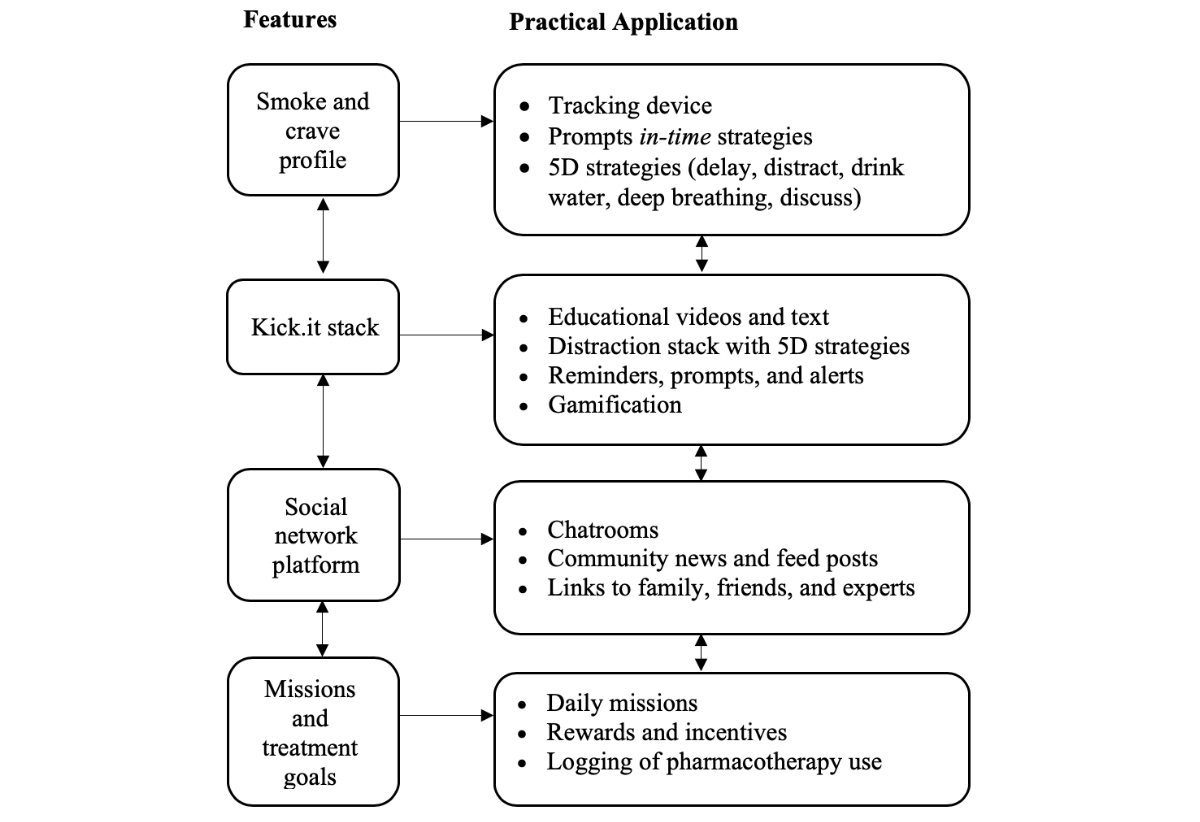
Kick.it app features and practical applications.

There are 4 core features contained in the Kick.it app. The *smoke and crave profile* feature is based on the principles of ecological momentary assessment [33], which tracks user’s smoking and quitting behaviors in real time and delivers *in-time* quit strategies at critical moments to prevent smoking relapse and support smoking cessation (see Multimedia Appendices 1 and 2 for screenshots of in-time interventions). When an app user logs a smoke or crave, it activates the tracking device and provides them with a progress report [28] (see Multimedia Appendix 3 for screenshot of the tracking device). The *Kick.it stack* feature contains education and strategies to assist app users during their quit attempt (see Multimedia Appendix 4 for screenshot of an educational video on nicotine replacement therapy). The *social network platform* is a unique app feature that leverages peer support and normative social influence for smoking cessation through chatrooms, community feeds, and links to other app users’ social networks [28]. To our knowledge, Kick.it is the first app to include a social network feature to enhance social support for SMI populations. The *missions and treatment goals* feature is based on an incentive and reward system that encourages app users to engage in daily health-enhancing activities and log their pharmacotherapy use. A comprehensive overview of the development and design of the Kick.it app has been published elsewhere [28]. Figure 1 presents the Kick.it app features and practical applications [28].

Assessing the feasibility, utility, and acceptability of tailoring the Kick.it app for SMI populations provides an ideal opportunity to address the limited availability of smoking cessation apps for this population.

### Study Aims

This study aimed to gain a deeper understanding of the lived experiences of smoking and quitting among people with SMI and their perception of social support for smoking cessation in relation to the Kick.it app. These experiences informed the researchers understanding of their perceptions regarding the feasibility, utility, and acceptability of the Kick.it app features to guide the tailoring of the app to their specific needs and preferences. The following research questions were investigated: (1) what are the facilitators and barriers to smoking cessation experienced by people with SMI and their perceptions of social support for smoking cessation? and (2) what features of the generic Kick.it app are perceived as feasible, useful, and acceptable in supporting people with SMI to prevent smoking relapse and quit smoking? [14].

## Methods

### Design

Co-design methodology was used for this qualitative inquiry because of its value in offering consumer involvement and collaboration [34] in the tailoring of the Kick.it app, which is well matched to meet the study aims [14]. Co-design methods included the triangulation of semistructured in-depth interviews, observation, and in-situ exploration [35] of the Kick.it prototype app with participants.

### Sample

Research ethics approval was obtained from the Southern Adelaide Clinical Human Research Ethics Committee (reference no. 16.17). A sample of people with SMI was then drawn from the community mental health services (CMHS) and the South Australian Cancer Council’s Quitline, within the Adelaide metropolitan area, Australia, between February 2018 and June 2018. Quitline is a free telephone service that provides information and advice to support people to quit smoking [36]. A purposive sampling method was used to recruit participants who were reflective of the target population and who meet the selection criteria [37]. CMHS and Quitline staff identified and screened potential participants’ eligibility to be involved in the study using the selection criteria. The inclusion criteria were (1) self-reported diagnosis of an SMI, (2) adult smokers (aged 18 years or more) who had attempted to quit smoking in the past 12 months and ex-smokers (abstinence for 7 days or longer before the interview) [38], and (3) the ability to provide informed consent as confirmed by CMHS or their doctor. The exclusion criteria were (1) individuals with acute severe suicidality or current acute psychosis as confirmed by CMHS or their doctor, (2) a sensory or motor impairment affecting the individual’s ability to participate in the study, and (3) a severe cognitive impairment affecting the individual’s ability to provide informed consent as confirmed by CMHS or their doctor [15]. Self-reported smoking status was determined using the following smoking status question “Which of the following best describes your smoking status?” and prompted from responses, “I’m a smoker, I smoke daily” and “I’m an ex-smoker, I never smoke now” [39].

CMHS and Quitline staff invited eligible participants to participate in the study and provided the contact details to the research team. Team member (PK) followed up with participants to further describe the study and organize an interview time. At the time of interview, written informed consent was obtained.

### Data Collection

Two consecutive semistructured in-depth interviews were conducted. This study included an iterative 2-staged interview approach to provide participants with an individualized experience [34]. This was particularly important during the stage 2 interviews, as it enabled people with SMI to receive the personalized assistance needed to navigate the app and the time to reflect and provide feedback on its features.

Stage 1 interviews (approximately 1 hour) consisted of open-ended questions to elicit rich data and a depth of understanding regarding participants’ smoking-related experiences. The interview guide was informed by the research questions, the relevant literature [40,41], and in consultation with the research team. Stage 2 interviews (approximately 1.5 hours) continued to explore participants’ smoking-related experiences in relation to the app. These sessions involved sitting with each participant, as they viewed the prototype app and asking questions in relation to their perception of its features in accordance with the stage 2 interview guide. Observations and field notes were also used to record any reflections on the interview process [37] and the participants’ ability to navigate the app [14]. The stage 2 interview guide was developed from review of the limited studies on tailoring smoking cessation apps for SMI populations [21,24,25], in consultation with the research team, and from preliminary analysis of the stage 1 interviews. The interview guides were reshaped somewhat in accordance with the iterative process of analyzing the data as it was being collected to enable a flexible approach that allows a review and refinement of the interview guide questions (eg, redundant questions were excluded) [37]. Table 1 gives examples of stage 1 and stage 2 interview guides. The interviews were audio recorded and transcribed verbatim by an accredited transcriber to preserve the meaning and authenticity of the participants’ responses. The transcribed interviews were then compared against the audio recordings to ensure their accuracy.

### Data Analysis

Thematic analysis was used as it provides a systematic approach to organizing, categorizing, and interpreting qualitative data [42,43]. The research team conducted open coding of the first 4 transcripts, independently of each other. In vivo coding was used to exemplify the meaning associated with participants’ responses [44]. A series of team meetings were then held to discuss and debate the initial codes and agree on a structure to guide the coding of the remaining interviews. Categories, selective codes, and emerging themes were captured in a spreadsheet and grouped to assist the researchers to gain a clearer sense of the themes emerging from the data [42-44]. Tables 2 and 3 give examples of stage 1 and stage 2 categories, selective codes, and participants’ frequency of responses.

An iterative process of reading and rereading the transcripts enabled the 3 researchers to reflect on and gain an in-depth understanding of participants’ stories [42,43]. Data were interpreted using a constant comparative approach within and between transcripts to help identify, review, and refine the codes and themes [45]. Mind maps were developed by ordering and linking the codes and categories to the themes. This process prompted robust debate among the researchers, which deepened the interpretation of meaning within the data and finalized the ordering of the themes [42,43]. A dualistic approach was used to utilize existing themes within the literature to build on the limited theory underpinning the design of smoking cessation apps for SMI populations (theory driven) and explore new emerging themes from this study’s findings (data driven) [37,46]. The triangulation of the different research team members’ interpretations and perspectives of the data added further methodological rigor [47]. Sample size was established according to evidence of data saturation being achieved [48]. The researchers concurred that data saturation was achieved by the sixth participant of the stage 2 interviews.

**Table 1:** Examples of stage 1 and stage 2 interview guides.

Interview guide examples^a^		Sample questions
**Stage 1**		
	Smoking behavior	How many years have you smoked cigarettes? What role does smoking play in your life?
	Smoking and mental health	How do you think smoking affects your mental health? What changes do you notice about your smoking when you are feeling psychologically unwell?
	Motivation to quit	How motivated are you to quit? What motivated you to quit smoking in the past?
	Quit smoking attempts	During your most recent attempt, what was it like for you to quit? How long did you quit for?
	Use of nicotine replacement therapy	Have you ever used nicotine replacement therapy to assist you to quit? What type of nicotine replacement therapy have you used?
**Stage 2**		
	App features	What do you think about the feature? What do you like/dislike about the feature?
	App content	How comfortable would you be sharing personal information with the app if it were to lead to a personalized quit program? What do you think about having content specific to mental illness and smoking?
	App functionality	Can you work out what to do to get to the next screen? What changes are needed to assist people with serious mental illness to work the app?
	App aesthetics	What do you think about the colors used in the app? What do you think about the font size? What do you think about the quality of the graphic images?
	Social support	What do you think about talking to other people on the app? What do you like/dislike about social media?

^a^Adapted from Vilardaga et al [21], Rotondi et al [24], Vilardaga et al [25], Rand Corporation [40], and Rae et al [41].

**Table 2:** Examples of stage 1 categories, codes, and participants’ frequency of responses (N=12).

Selective codes		Statistics, n (%)
**Smoking behavior and experiences**		
	Smoking to manage mental illness/symptoms	12 (100)
	Nicotine addiction	12 (100)
	Stigma associated with smoking/mental illness	5 (42)
	Increased smoking consumption when unwell	12 (100)
	Self-esteem/self-efficacy	10 (83)
**Effects of smoking on mental health**		
	Perceived benefits of smoking	12 (100)
	Aware of adverse effects of smoking	5 (42)
**Triggers for smoking**		
	Withdrawals/cravings	11 (92)
	Smoking and mental health	12 (100)
	Smoking to manage life events/stressors	8 (67)
**Quitting behavior and experiences**		
	Mental illness and smoking relapse	9 (75)
	Difficulty managing withdrawals	10 (83)
	Coping with cravings	11 (92)
**Nicotine replacement therapy**		
	Use of nicotine replacement therapy	9 (75)
	Never used nicotine replacement therapy	3 (25)
	Positives associated with use	7 (58)
	Adverse side effects of use	6 (50)
**Perceived benefits of quitting**		
	Saving money	12 (100)
	Improved health	12 (100)
**Barriers to quitting**		
	Mental illness	11 (92)
	Coping with cravings	11 (92)
	Stress-related factors	10 (83)
**Perception of social support**		
	Use of social supports	10 (83)
	Reluctance to access	2 (17)
**Use of app/Web-based resources**		
	Smoking cessation apps	3 (25)
	Health apps	5 (42)
	Other apps (eg, weather)	9 (75)
	Never used apps	3 (25)
	Social media (eg, Facebook)	8 (67)

**Table 3:** Examples of stage 2 categories, codes, and frequency of participants’ responses (N=12).

Selective codes		Statistics, n (%)
**App tailored to app users’ needs**		
	Creates a profile based on mental illness and smoking	12 (100)
	Develops a personalized quit smoking program	12 (100)
	Tailored strategies specific to mental illness/addiction	10 (83)
**Smoking relapse**		
	App reassures that quitting can take numerous attempts	6 (50)
	App encourages rapid return to quitting	5 (42)
**An empathetic app**		
	Uses empathetic/positive communication that looks after self-esteem	6 (50)
**Social network**		
	Enhance social/peer support for smoking cessation	10 (83)
	Perceived utility of a social network for smoking cessation	8 (67)
	Acceptability of a social network for smoking cessation	10 (83)
	Contingency plan to manage risks/privacy	4 (33)
	Contains chatrooms specific to mental illness and smoking	9 (75)
	Reduces stigma, social isolation, and loneliness	7 (58)
**Kick.it app features**		
	Utility, usefulness, and acceptability of app features	12 (100)
	Most useful features	12 (100)
	Least useful features	9 (75)
**App functionality**		
	Able to navigate the app without assistance	10 (83)
	Difficulty navigating the app without assistance (ie, observed usability issues associated with working the app, confirmed lack of experience using smartphones/apps)	2 (17)
**App aesthetics**		
	Colors, font size, and quality of the graphic images	8 (67)

## Results

### Overview

A total of 12 adults with SMI participated in the study, comprising 6 male and 1 female smokers, and 2 male and 3 female ex-smokers. All participants had been medically diagnosed with either an individual diagnosis of schizophrenia, borderline personality disorder or bipolar disorder, or psychiatric comorbidity. Most participants (75%, 9/12) were diagnosed with paranoid schizophrenia and psychiatric comorbidities, such as depression and anxiety. Some participants (58%, 7/12) had a socioeconomic disadvantaged status, as indicative of these participants’ receiving disability support pension as their primary source of income. All participants were in receipt of community-based support services. Participants’ characteristics are presented in Table 4.

Key findings highlighted several psychosocial factors as important in tailoring the Kick.it app for SMI populations. The key themes that emerged from the data in relation to participants’ lived experiences of smoking and quitting and their perceptions of the Kick.it app features are described below. Key findings aligned with broader psychosocial needs and experiences of perceived stigma and social isolation for this population, which indicated that smoking cessation efforts are inseparable from the environmental and personal context in which these smokers experience and cope with mental illness in their community. Examples of participants’ quotes that help to exemplify the meaning and interpretation of participants’ responses are also included. Multimedia Appendix 5 gives more examples of participants’ quotes.

**Table 4:** Characteristics of participants with serious mental illness (N=12).

Characteristics		Statistics, n (%)
Age (years), range (median)		31-53 (47.5)
**Gender**	
	Male	8 (67)
	Female	4 (33)
**Smoking status**	
	Current smoker	7 (58)
	Ex-smoker	5 (42)
**Smoking behavior**	
	Heavy smoker (>20, daily)	12 (100)
	Years smoked, mean (SD)	26 (12.3)
	Cigarettes smoked per day, mean (SD)	28 (9.9)
**Primary psychiatric diagnosis**	
	Schizophrenia disorder	9 (75)
	Borderline personality disorder	2 (17)
	Bipolar disorder	1 (8)
**Psychiatric comorbidities (n=7)^a^**	
	Anxiety	7 (100)
	Depression	4 (57)
	Schizoaffective disorder	1 (14)
	Posttraumatic stress disorder	1 (14)
**Level of education**																							
	Tertiary education	2 (17)
	Technical and Further Education	4 (33)
	High school	6 (50)
**Source of income**	
	Full-time work	2 (17)
	Part-time work	2 (17)
	Disability support pension	7 (58)
	Other	1 (8)
**Marital status**	
	Single	7 (58)
	Partnered	4 (33)
	Divorced	1 (8)
**Type of residence**	
	Supported residential facility	2 (17)
	Independent living	10 (83)
	Living alone	4 (33)
	Living with others	6 (50)
**Quit attempts**	
	Single attempt	3 (25)
	Multiple attempts	9 (75)
**Use of social support resources**	
	Family and friends	8 (67)
	General practitioner	4 (33)
	Mental health caseworker	2 (17)
	Quitline call center	2 (17)
	Nicotine replacement therapy	9 (75)
	Smoking cessation apps	3 (25)
	Smartphone ownership	9 (75)
	Use of social media	8 (67)

^a^A total of 7 participants presented with psychiatric comorbidities.

### Special Needs

#### An App That Tailors a Personalized Quit Program to an Individual’s Psychosocial Needs

Exploration of the participants’ perception of the Kick.it app features highlighted the importance of the app tailoring a personalized quit program to their needs. This included the app tailoring a program specific to their psychiatric diagnoses (and consequent symptoms) and smoking behavior. Participants described their smoking behavior as a form of self-medication as it provides them with a source of comfort to relieve their symptoms of mental illness:

...an app that’s tailored to mental health consumers is essential...if it’s generic it won’t delve into the personal struggles that they’re going through with having to look at smokes as being their only source of comfort.

interview session (IS) 1, participant (P) 1

All participants indicated that their smoking consumption almost doubled when they were feeling psychologically unwell, which reflected their reliance on cigarettes to help them to cope with their mental illness:

When I’m depressed...I just would like to be left alone with my cigarettes and coffee...[smoking] goes up to about 40 a day.

IS1, P3

Some participants (83%, 10/12) also indicated that stressful social environments, such as relationship problems, peer smoking, and work-related issues, were barriers to smoking cessation:

I use it as a stress reliever...where I’m completely thinking of nothing else other than smoking...I’m not worried about uni, family or work problems...

IS1, P7

It was evident from listening to participants stories that people with SMI have many psychosocial issues and need support to manage their mental illness, nicotine dependency, and social-related issues while attempting to quit (see Multimedia Appendix 5 for more quotes relating to this theme).

#### An App That Normalizes Smoking Relapse and Multiple Quit Attempts

Exploration of participants’ quitting experiences revealed that most participants had attempted to quit on several occasions (75%, 9/12), but their attempts were often short lived. For example, some participants’ recalled occasions where they were determined to quit, but within a few hours when the intensity of the cravings had occurred, they were reaching for a cigarette:

I have [tried to quit] many times. Two hours later I’ve got a fag in me hand.

IS1, P3

Participants described overwhelming feelings of disappointment and helplessness regarding their ability to sustain a quit attempt. These findings indicated that an app tailored to support people with SMI may focus more on reassuring them that smoking relapse is a normal part of the quitting process, and that it can take numerous attempts to quit:

When you relapse you’re disappointed with yourself and you smoke more than you did before...the best thing about an app that reassures you is that...it’s okay to have the relapse but get back on the bandwagon...try the app again.

IS2, P1

Our findings also indicated that standard smoking cessation approaches that require a range of cognitions such as critical and analytical thinking, evaluating, judging, and weighing options, and deciding on actions that can foreground planning to quit caused participants heightened anxiety and stress. Participants reported that feelings of anxiety and stress increased their smoking consumption and were major barriers to smoking cessation. Therefore, asking people with SMI to recall quit strategies in those moments when they are feeling anxious and experiencing intense withdrawals offer limited smoking cessation support. These findings indicated that there is a need for alternative smoking cessation approaches that address these temporal issues by assisting people with SMI to quit smoking in real time, within the context of their daily lives:

If you can offer practical solutions for people to try in certain situations...that would be a much better deterrent to lighting up.

IS2, P7

Participants were impressed with the smoke and crave profile generated in the Kick.it app. They perceived the *in-time* quit strategy messages that app users receive when they log a smoke or crave as useful and acceptable in supporting them to quit. They also liked the tracking device as it would provide them with ongoing feedback regarding their smoking and quitting behaviors (see Multimedia Appendix 5):

Might be doing something then all of a sudden you get a message and you think...I’ll give that a try.

IS2, P9

#### Strong Focus on User Experience to Improve Usability of the App

Findings suggest the need to apply an optimal UX design through simple user interfaces [27] such as directional cues with arrows indicating to *swipe here* to improve usability of the app among people with SMI:

I didn’t know what to do when I was sliding across...you’re going to need a sign to say slide across here. It has to be really basic for people who are mentally ill.

IS2, P6

Furthermore, 2 participants with schizophrenia found navigating the app overwhelming as they possessed limited knowledge and skills in technology. Therefore, applying a simple app design increases the likelihood that people with SMI will be able to use the adapted Kick.it app:

What is it, an app? What does that mean? A phone? Email, it’s got internet on it? I’m not really quite sure what’s going on.

IS2, P2

Participants also reported that they appreciated our co-design approach as it enabled them to share their smoking-related stories and provide input on the tailoring of the app for SMI populations (see Multimedia Appendix 5):

The fact that you are interviewing me and other people with different experiences...you are making it [the app] really consumer-focused.

IS2, P12

### Preferences

#### A Caring App

Participants wanted a caring app, with almost human-like qualities that could offer companionship and enable them to share their concerns without feeling stigmatized or judged:

...if they get the idea that people actually care about the cigarette smoking...that people actually care for their health.

IS2, P3

The need for a caring app seemed to stem from the interplay between SMI, smoking, and stigma that featured heavily in both interviews and were common experiences among all participants. For example, some participants talked about schizophrenia being less accepted in the community than depression, which resulted in them experiencing social isolation associated with not having friends. Some participants also indicated that stigma was a major force driving their smoking behavior:

...people with schizophrenia get pushed away, and that’s why they get into their circles...smoking cigarettes...there’s a lot of stigma especially with things like schizophrenia.

IS2, P3

In addition, participants perceived that a caring app could contain messages that motivated them to quit, to believe in themselves, and that gave them hope that they could quit smoking (see Multimedia Appendix 5):

It’s nice to receive a positive statement because it’s quite daunting quitting, and you feel quite alone and isolated, like can I do this.

IS2, P7

#### A Social Network–Based App

Most participants (83%, 10/12) were enthusiastic about engaging with a social network. They liked the idea of having chatrooms specific to mental illness and smoking where they could connect with likeminded people who also wanted to quit. By enhancing peer support for smoking cessation through the social network function, the app has the potential to address stigma, social isolation, and loneliness. People with SMI can use their phone anytime and anywhere to connect with other people who are also using the app:

What’s good about it is following each other and giving each other support...they can interact with each other, because it’s important.

IS2, P5

In relation to privacy and confidentially, most participants liked the inclusion of *terms and conditions* that outlined the *privacy settings* and *rules of use* to alleviate potential concerns around engaging with the social network (see Multimedia Appendix 5):

...it’s bound by privacy so you know you can talk about this issue...and it’s just the community that you’re working on this issue with...it’s not going out to everybody.

IS2, P12

#### Social Support Resources for Smoking Cessation

Exploration of participants’ perception and utility of social support for smoking cessation indicated that most participants received social support (83%, 10/12) from their family, friends, and general practitioner:

I’d planned to give up smoking with a friend...mum was supportive...

IS1, P4

Many participants (75%, 9/12) reported using nicotine replacement therapy to assist their quit attempts, but some participants had experienced adverse physical and/or mental health effects (50%, 6/12), which deterred them from continuing its use:

...you still feel like smoking on nicotine replacement therapy, but the cravings are not as bad, you don’t get as agitated without smokes.

IS1, P8

Overall, 3 participants reported that they had used a smoking cessation app to support a quit attempt. Of these, 2 participants found the app useful in supporting their attempt. It is often assumed that younger people are more likely to use apps; however, this finding provided some insight into the use of smoking cessation apps among adults aged 36 to 52 years, with SMI (see Multimedia Appendix 5):

It’s [the app] just something that’s always there...it was there for me at the touch of a phone.

IS1, P4

**Figure 2:**
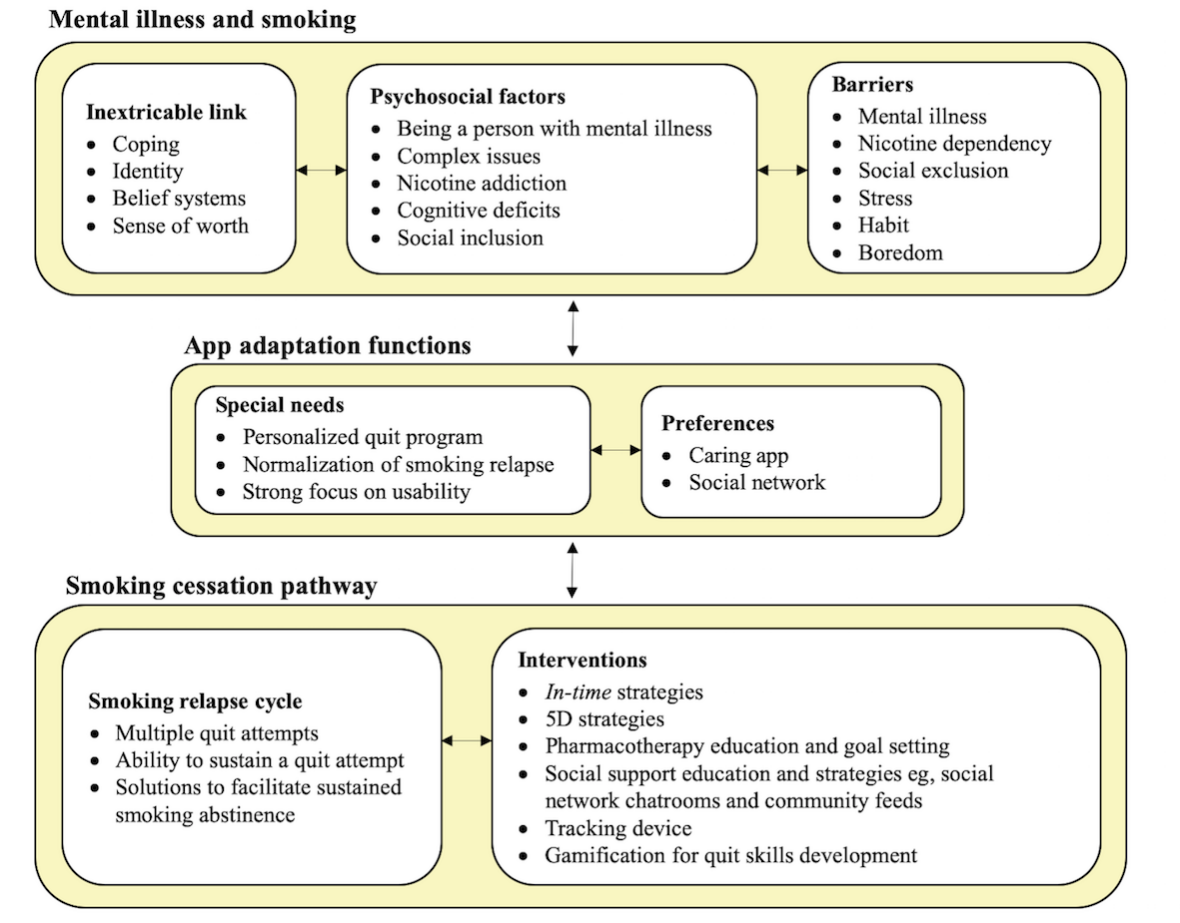
Key findings guiding the tailoring of the Kick.it app for serious mental illness populations.

The key findings guiding the tailoring of the Kick.it app for SMI populations, including participants’ lived experiences of smoking and quitting, their identified needs and preferences for tailoring the Kick.it app, and the smoking cessation pathway outlining the smoking relapse cycle and smoking cessation intervention, are illustrated in Figure 2.

## Discussion

### Principal Findings and Comparison With Previous Work

Results suggest that tailoring of the Kick.it app is feasible, useful, and acceptable for people with SMI. Participants were interested in using mHealth interventions to support their quit smoking efforts. Similar to other studies [21,25], our findings indicated using a co-design approach to improve the utility, usability, and acceptability of smoking cessation apps for SMI populations is required.

Exploring participants’ smoking and quitting experiences highlighted the complexity associated with the inextricable link between mental illness and smoking [6,7], which has pervasive impacts on participants’ psychosocial well-being and affects every aspect of their daily lives. These findings suggest that specific tailoring of the app is needed to assist people with SMI to navigate the complex interaction between mental illness and smoking that impact on their capacity to quit. Consistent with previous evidence [10], results of this study found that current smoking cessation approaches are limited in supporting people with SMI to quit as they do not account for their mental health–related needs [49], nor do they address the symptoms of mental illness and nicotine dependency simultaneously. The need for a dual approach to smoking cessation that provides quit strategies to address both the symptoms associated with smokers’ psychiatric diagnoses and nicotine addiction was identified many years ago [50]. Another limitation of current smoking cessation approaches is that they do not offer *real-time* assistance regarding experiencing the urge to smoke. For example, they do not address stressors arising in the context of their daily lives in the moments [33] when those stressors are heightened, and the person is at high risk of reaching for a cigarette to alleviate their distress. The delivery of novel smoking cessation approaches that offer assistance in the *here and now* to support people with SMI to prevent smoking relapse and quit smoking has been established as important in this study and warrants further investigation. The Kick.it app’s smoking cessation interventions may provide the solution to address the limitations of current smoking cessation approaches and support people with SMI to quit.

This study also provided valuable insight into the effects of stigma and a possible solution to the perpetuated entrenched marginalization and social disadvantage among smokers with SMI [11,49]. Gaining a deeper understanding of participants’ lived experiences of stigma and how those experiences had impacted on their self-worth highlighted why it was important to them to have a caring app. Therefore, the features of the Kick.it app may be different from generic smoking cessation apps in that it could focus more on building their self-esteem and self-efficacy. There is also potential for the Kick.it app’s *caring* features and social network to reduce the effects of stigma, social isolation, and loneliness by enhancing social inclusion, and a sense of belonging to a social support network for smoking cessation. The benefits of using a social network to gain peer support for smoking cessation among people with psychosis has been established [51].

An adapted Kick.it app has the potential to become an all-encompassing solution, a virtual friend that offers around the clock support to help people with SMI to quit, and address stigma which is a by-product of SMI [49]. The specific app features and their adaption that participants perceived would be feasible, useful, and acceptable in assisting SMI populations to quit smoking included the smoke and crave profile, the supportive messaging, the tracking device, and social networking.

### Limitations

Limitations of the study included the participants’ self-reported [38] smoking status and psychiatric diagnoses. However, smoking status was obtained using a screening tool adapted for this population [39], and all but 1 participant was recruited from CMHS, which provides ongoing case management support to people with existing SMI. Another limitation relates to the participants’ sampling a *prototype* of the Kick.it app at the stage 2 interviews which provided users with a brief window of 1.5 hours to view the app and advise on its features in an interview setting rather than a naturalistic setting [37].

### Conclusions

This study provides evidence for innovative smoking cessation approaches to support people with SMI to prevent smoking relapse and successfully quit. We contributed to the limited knowledge on designing smoking cessation apps for SMI populations by using a co-design principal based on the IM framework to explore their lived experiences of smoking and quitting and their perception of the Kick.it app features to guide the tailoring of the app. Through the lens of people with SMI, this study provides insight into the smoking behaviors and personal struggles they encounter in their endeavor to quit smoking. By confronting some of the major barriers to smoking cessation for this population, this study contributes to possible solutions for important mental health–related issues, including stigmatization and social isolation [11,49]. The next stage of research planned by the authors of this study involves tailoring the Kick.it app in accordance with the findings and then conducting a quantitative study to gain a representative sample to assess the effectiveness, utility, and acceptability of the app among people with SMI in relation to smoking cessation [14].

## Appendix

Multimedia Appendix 1 Screenshot of a social support in-time intervention.

Multimedia Appendix 2 Screenshot of a 5D strategy in-time intervention.

Multimedia Appendix 3 Screenshot of the tracking device.

Multimedia Appendix 4 Screenshot of an educational video on nicotine replacement therapy.

Multimedia Appendix 5 Key themes and participant quotes.

## Abbreviations

CMHS: community mental health services
IM: intervention mapping
mHealth: mobile health
SMI: serious mental illness
UX: user experience
