# Supplementary figures and images for: Tailoring of a Smartphone Smoking Cessation App (Kick.it) for Serious Mental Illness Populations: Qualitative Study
Source: JMIR Hum Factors. 2019 Sep 3;6(3):e14023. doi: 10.2196/14023 (PMC6754228; doi:10.2196/14023)

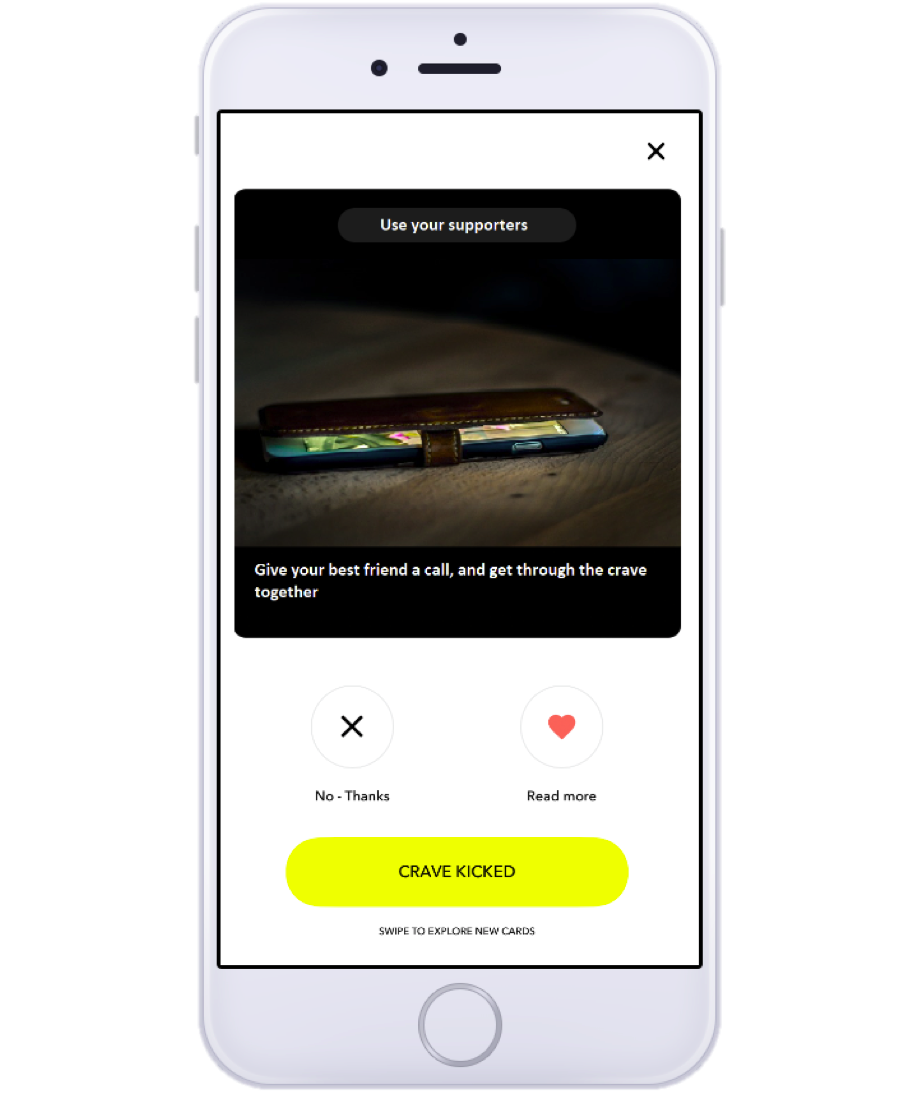

Supplement: Multimedia Appendix 1 [file humanfactors_v6i3e14023_app1.png]

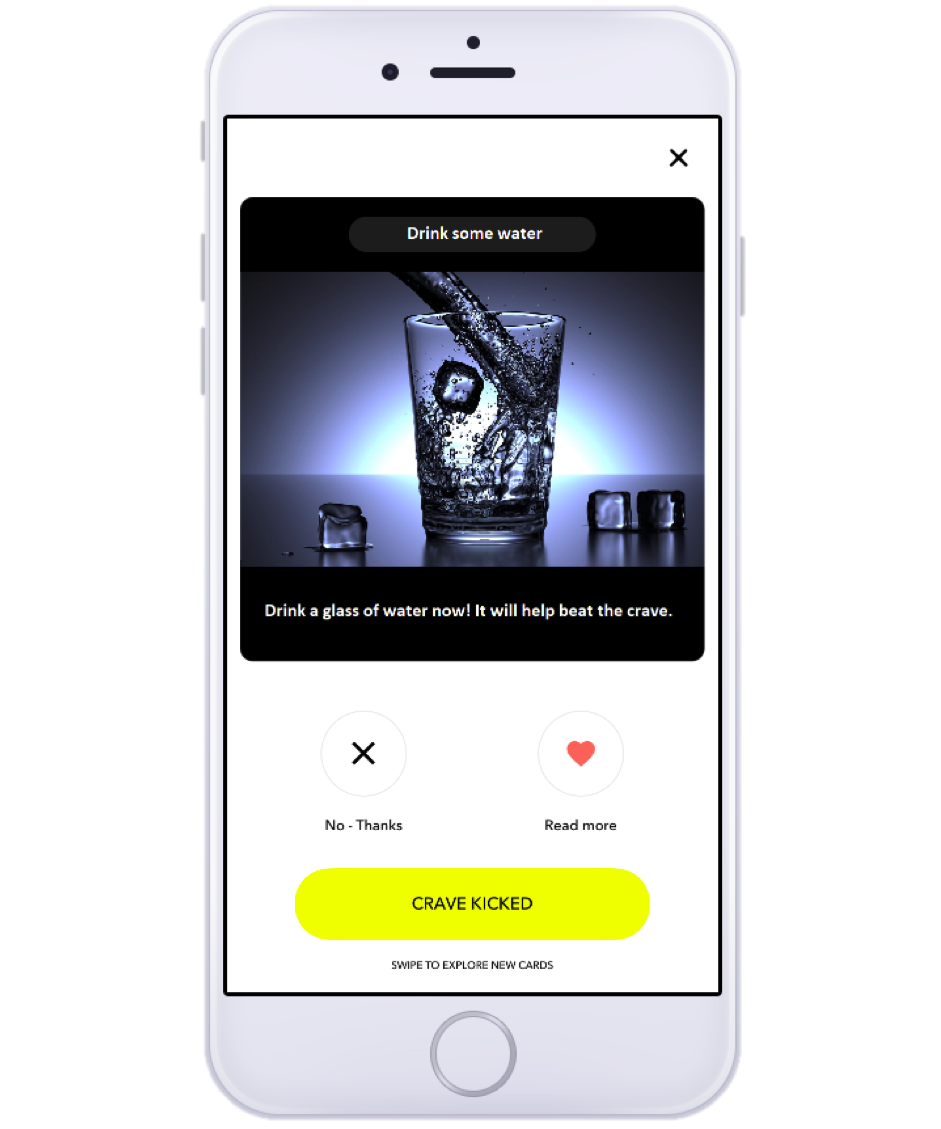

Supplement: Multimedia Appendix 2 [file humanfactors_v6i3e14023_app2.png]

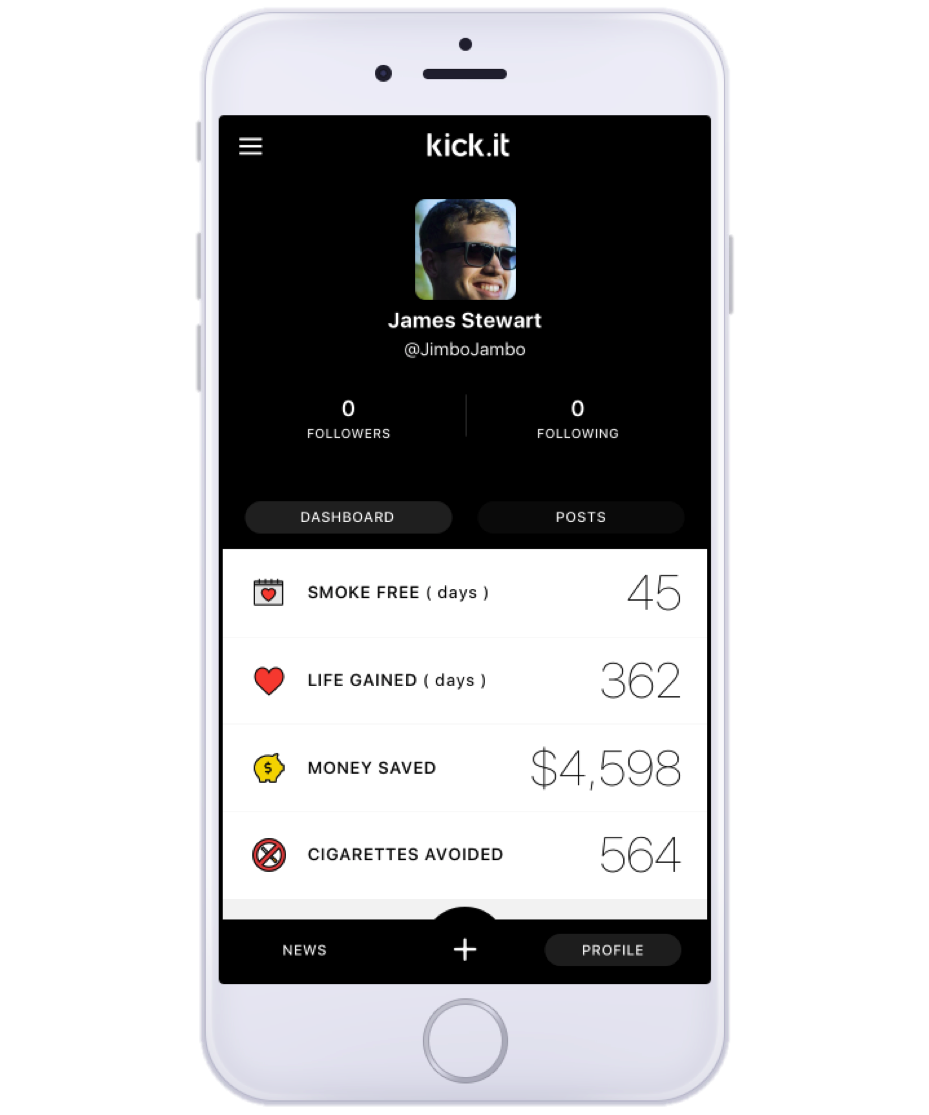

Supplement: Multimedia Appendix 3 [file humanfactors_v6i3e14023_app3.png]

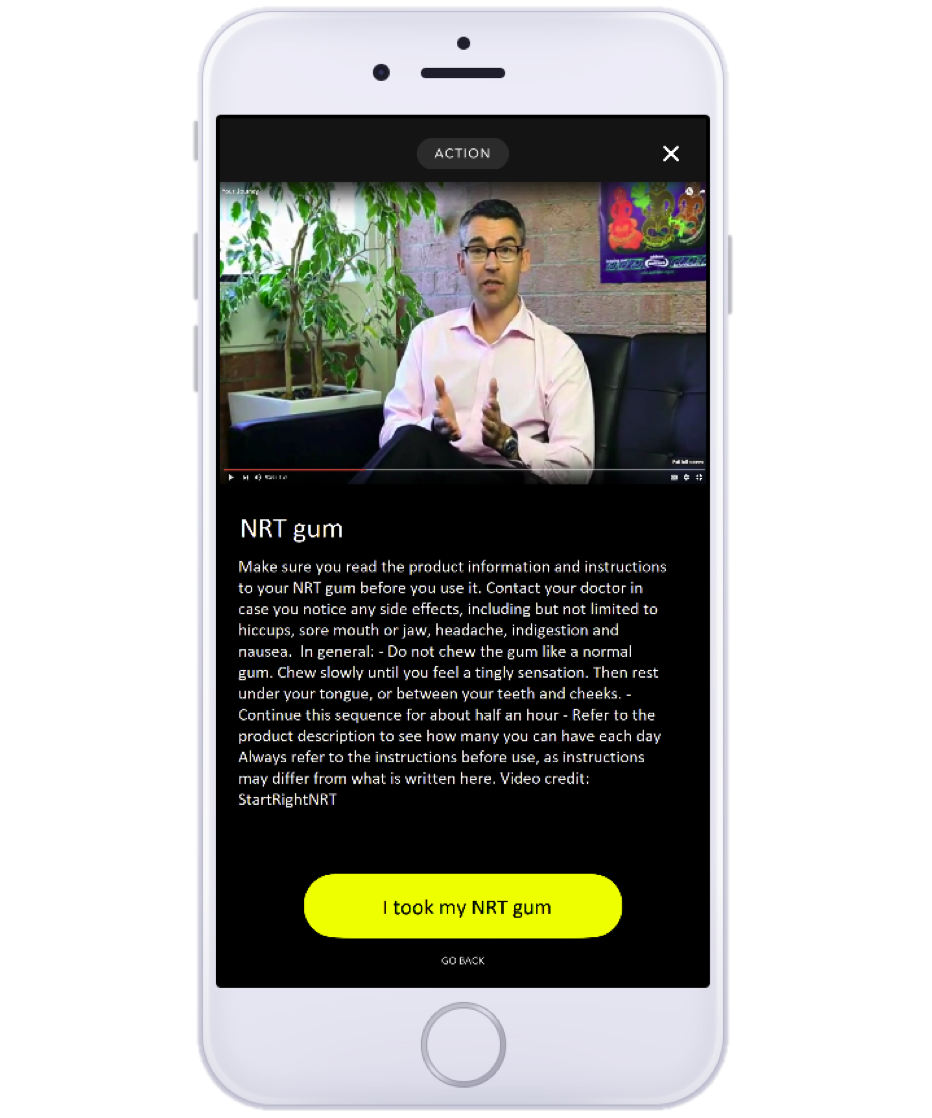

Supplement: Multimedia Appendix 4 [file humanfactors_v6i3e14023_app4.png]
