# Supplementary material for: Tailoring of a Smartphone Smoking Cessation App (Kick.it) for Serious Mental Illness Populations: Qualitative Study
Source: JMIR Hum Factors. 2019 Sep 3;6(3):e14023. doi: 10.2196/14023 (PMC6754228; doi:10.2196/14023)
Supplement: Multimedia Appendix 5 [file humanfactors_v6i3e14023_app5.pdf]

Multimedia Appendix 5. Key themes and example participant' quotes

Themes, participant quotes	
**An app that tailors a personalized quit program to an individual's psychosocial needs**	
	It's good to have a unique profile...everyone's different so you've got to tailor it to each person. [IS2, P11]
	I think one tailored for mental health patients is a good idea because it's more specific. [IS2, 08]
	...it would be really useful if it had topics on schizoaffective disorder, depression, anxiety, if you're feeling anxious, [quit] strategies for being anxious. If they're experiencing auditory hallucinations they have a tendency to smoke a lot... They could go to the app...and use those strategies instead of smoking. [IS2, P1]
	...people with mental illness if they've been smoking a long time they see it as a remedy to help calm their feelings, for example, when I'm anxious I smoke a lot and that just gives me a brief reprieve. [IS1, P1]
	...it [smoking] is sort of like a comfort thing for me... [IS2, P7]
	Just the awful feeling physically [withdrawals]. I need the nicotine so that would make me feel less insane. [IS1, P2]
**An app that normalizes smoking relapse and multiple quit attempts**	
	I can't give up, I've tried everything! [IS1, P1]
	...I always fail when I plan a date...and every time I do, I end up smoking more and more before I get to the date, and then when I get to the date, it's all too hard. [IS2, P1]
	Stress, anxiety, when I smoked I felt a lot calmer. [IS1, P5]
	Smoke free days, so someone might have been trying to quit smoking...they can go wow, in that time there have been 45 days that I haven't [smoked] and that would be really positive. [IS2, P12]
	Keeping track of progress...if you can see where you've come, that can give you more incentive to give up. [IS2, P7]
	Yeah, that'd be interesting... just a visualisation you know, of what's happened. [IS2, P9]
**Strong focus on user experience to improve usability of the app**	
	[Trying to work the app] I don't understand. Oh, like that? And swipe it? [IS2, P10]
	The main thing is the app is easy use...quick, simple, that's what people want this day and age with technology. [IS2, P5]
	I think with things like this [the app], the simpler the better for a user... [IS2, P7]
	[Colours used in the app] I'd change it. Maybe a white background with the yellow writing or something. Is there a homepage? That's something they've got to look at too once it's live. You could have a fist...a crushed fist with a cigarette in your hand... Breaking the habit. I mean, picture like – if someone's got a good hand with a cigarette... And literally have break the habit written down there... [IS2, P5].
	I would be putting this swipe left in the tiny letters, at least you can see it and it's consistent with what you should do. I mean some might just get it, but it might, yeah need to be on each of the different changes [slides] ...the size of the font's fine. I think it's not too big and it's not too small. I'm just trying to consider

	someone with glasses... [IS2, P7].
	[Font size] Well I need to wear glasses these days, I couldn't see it otherwise. I think it's good because I think most people are either wearing glasses or have good enough vision. And people also can adjust the settings on their phone to make it bigger or smaller. It [the app] actually looks really good and it's got a hopeful feel to it as well. [IS2, P12].
	[Codesign approach] The fact that you've come to me is a major step, and today has made me feel that what I have done [quit] is actually worth it. It's validated everything that I've done. [IS1, P4]
**A caring app**	
	I would have liked to have felt that this app was designed for me...that I was being looked after as a person with a mental illness... [IS2, P4]
	...a lot of that stigma, it was soul destroying, you felt like...you're less worthy than others in the world... A lot of that stuff drove the cigarettes. [IS1, P7]
	Most mentally ill people don't have many best friends, and some of them don't have any friends at all. [IS2, P6]
**A social network-based app**	
	...I can't speak for all people with mental illness, but, it's a lonely life, really it is...if you can crack the loneliness. [IS1, P3]
	One of my problems is the social aspect where a lot of the people I know smoke...I feel like I'm missing out so I start smoking again... [It's] the sense of loneliness. [IS1, P8]
	It's good to know that I'm not alone and you're covered 24/7 if you wake up at 3 a.m. and you're dying for a cigarette...you've got your phone, it's there. [IS2, P11]
	[Chatrooms specific to mental illness and smoking] That would be very good because then they would know that their not on their own, that their not the only one doing this...whereas, most people that are normal type thing, they have their friend list and stuff like that, but it would be very hard for a person with schizophrenia to converse with someone in a lot of ways that does not have schizophrenia... [IS2, P3]
	...you've got the privacy settings that they can set so it's safe. [IS2, P11]
**Social support resources for smoking cessation**	
	I didn't really have any support, didn't ask for it, I just did it, bar mum... (IS1, P6)
	It's mainly my GP that I felt the most confident speaking to. [IS1, P7]
	I used the Quit app...it was good, it reaffirmed a lot... [IS1, P7]
	I've tried the inhaler, I've tried patches, I've tried lozenges, the big ones and the little ones, and in the end, you start doing anything you can to avoid using them. [IS1, P4]
